# Increment of Maternal Mortality Among Admissions for Childbirth in Low-risk Pregnant Women in Brazil: Effect of COVID-19 Pandemic?

**DOI:** 10.1055/s-0042-1751059

**Published:** 2022-07-07

**Authors:** Bruna Depieri Michels, Daniela Ferreira D'Agostini Marin, Betine Pinto Moehlecke Iser

**Affiliations:** 1 Faculty of Medicine, Universidade do Sul de Santa Catarina, Tubarão, SC, Brazil; 2 Post Graduate Program in Health Sciences, Universidade do Sul de Santa Catarina, Tubarão, SC, Brazil

**Keywords:** mortality, maternal, COVID-19, postpartum period, health impact assessment, mortalidade materna, COVID-19, período pós-parto, avaliação do impacto na saúde

## Abstract

**Objective** To assess the possible impact of the COVID-19 pandemic on maternal mortality among admissions for childbirth in 2020 in relation of the last 10 years.

**Methods** An ecological study with pregnant women who underwent hospital births at the Brazilian unified public health service (SUS, in the Portuguese acronym) in Brazil from 2010 to 2020. The mortality among admissions for childbirth was obtained based on the number of admissions for childbirth with reported death as outcome divided by the total number of admissions. The underlying gestational risk and route of delivery were considered based on the national surveillance system. The average mortality for the period between 2010 and 2019 (baseline) was compared with the rate of deaths in 2020 (1 ^st^ pandemic year); the rate ratio was interpreted as the risk of death in 2020 in relation to the average of the previous period (RR), with 95% confidence intervals (CIs).

**Results** In 2020, the 1 ^st^ year of the COVID-19 pandemic, 1,821,775 pregnant women were hospitalized for childbirth and 651 deaths were reported, which represents 8.7% of the total hospitalizations and 11.3% of maternal deaths between 2010 and 2020. There was an increase in maternal mortality after births in 2020 compared with the average for the period between 2010 and 2019, specially in low-risk pregnancies, both in vaginal (RR = 1.60; 95%CI:1.39–1.85) and cesarean births (RR = 1.18; 95%CI:1.04–1.34).

**Conclusion** Maternal mortality among admissions for childbirth according to SUS data increased in 2020 compared with the average between 2010 and 2019, with an increment of 40% in low-risk pregnancies. The increase was of 18% after cesarean section and of 60% after vaginal delivery.

## Resumo

**Objetivo** Avaliar os possíveis impactos da pandemia de COVID-19 na mortalidade materna nas admissões para o parto em 2020 em relação ao histórico dos últimos 10 anos.

**Métodos** Estudo ecológico com gestantes que realizaram parto hospitalar pelo Sistema Unificado de Saúde do Brasil (SUS) de 2010 a 2020. Para obter-se a taxa de mortalidade entre as admissões para o parto, foi utilizado o número de internações para parto que tiveram óbito como desfecho dividido pelo total de internações. O risco gestacional e o tipo de parto foram considerados a partir do sistema de vigilância nacional. A média de mortalidade no período de 2010 a 2019 (linha de base) foi comparada com a taxa de mortalidade pós-parto de 2020 (1° ano pandêmico); a razão das taxas foi interpretada como risco de óbito em 2020 em relação à média no período anterior (RR), com intervalo de confiança (IC) de 95%.

**Resultados** Em 2020, 1° ano da pandemia de COVID-19, 1.821.775 gestantes foram internadas para o parto e 651 óbitos foram registrados, o que representa 8,7% do total de internações e 11,3% das mortes maternas entre 2010 e 2020. Houve aumento na mortalidade materna após partos em 2020 em relação à média do período entre 2010 e 2019, especialmente em gestações de baixo risco, tanto em partos normais (RR = 1.60; IC95%: 1.39–185) quanto em cesáreas (RR = 1.18; IC95%: 1.04–1.34).

**Conclusão** A mortalidade entre as admissões para o parto pelo SUS aumentou em 2020 em relação à média de óbitos entre 2010 e 2019, com um incremento de 40% em mulheres de baixo risco gestacional. O aumento verificado foi de 18% após cesárea e de 60% após parto vaginal.

## Introduction

Maternal mortality is a profound violation of the human rights of women, mainly because it is considered preventable in 92% of the cases. 1 Thus, reducing maternal mortality is one of the goals of the World Health Organization (WHO) sustainable development goals for 2030. 2

In 2020, the Sars-CoV-2 pandemic showed up as a new obstacle to ensuring maternal and fetal health: pregnant and postpartum women were considered an infection risk group for developing more serious complications. 3

In the same year, a Brazilian study found a12.7% lethality rate due to infection in the obstetric population, higher than in other countries. 4 5 6 7 In June 2020, 5 months after the 1 ^st^ case of COVID-19 in the country, the number of mothers who lost their lives due to the disease already represented 10% of the total annual maternal deaths. 4 Throughout the year, 453 deaths were registered, with a weekly average of 10.5 deaths. 8

In addition, 4,245 pregnant and postpartum women were infected by COVID-19 in 2020, and 352 of them (7.7%) died. When considering only the gestational period, 3,459 infections and 221 deaths were registered, recording 6.3% of deaths. However, when only puerperal women were considered, 786 infections and 131 deaths were registered, reaching a higher rate (14.3% of the patients died), which suggests that the puerperal period is more lethal than the gestational period. 8

It is important to emphasize that a large proportion of pregnant and postpartum women who died from COVID-19 infection did not have comorbidities or risk factors; in other words, they did not fit the definition of high-risk pregnancies, 9 when the life or health of the pregnant woman or of the fetus are more likely to be affected than those of the considered population. 10

Considering the effects of COVID-19 in the Brazilian obstetric population, the present study aims to assess the possible impact of the pandemic on maternal mortality among admissions for childbirth in 2020 in relation to the history of the last 10 years, according to the gestational risk and route of delivery in Brazil.

## Methods

This is a quantitative ecological study. The study population consisted of pregnant women who underwent hospital births at the Brazilian Unified Health System (SUS, in the Portuguese acronym), registered in the Hospital Information System (SIH/SUS, in the Portuguese acronym), in the period from 2010 to 2020. Complete data were obtained from health information available in the database of the Information Technology Department of the Brazilian Public Healthcare System (DATASUS, in the Portuguese acronym). Data were exported to Microsoft Excel (Microsoft Corporation, Redmond, WA, USA) and were analyzed using the software Stata version 12.0 (StataCorp, College Station, TX, USA).

The population of interest for the study was pregnant women who underwent hospital births at the SUS. The mortality among admissions for childbirth was obtained based on the number of admissions for childbirth who had maternal death as the outcome divided by the total number of admissions, for each route of delivery, gestational risk, and year of analysis. We used a 10 ^4^ constant. The average mortality for the period between 2010 and 2019 was compared with the maternal mortality rate of 2020. The gestational risk and route of delivery were classified, as recorded at the SIH, in: high-risk vaginal delivery; low-risk vaginal delivery; high-risk cesarean delivery; and low-risk cesarean delivery. This categorization is recorded in the system by the health professional responsible for the assistance, considering the definition of high or low gestational risk. 10

To analyze a possible effect of COVID-19 on maternal mortality, the period from 2010 to 2019 was considered the baseline, and the year 2020, because of the introduction of COVID-19, the exposure period. So, to measure the impact of 2020 and, indirectly, of the pandemic on mortality among admissions for childbirth, the average mortality in the period between 2010 and 2019 (considered nonexposed period rates) was compared with the mortality presented in 2020 (1 ^st^ year of the pandemic, considered the exposed period). The rate ratio (RR) was interpreted as the risk of death in 2020 in relation to the average of the previous period. Confidence intervals (CIs) of 95% were estimated, with a significance level of 5%. The analyses were carried out using the Stata statistical program package version 12.0 (StataCorp, College Station, TX, USA) and OpenEpi.

All data is public and available on the Internet with unrestricted access data and without identifying individuals. The present publication is part of a project approved by the Ethical Review Board (No. 4.482.150 of December 2020).

## Results

**Fig. 1.**
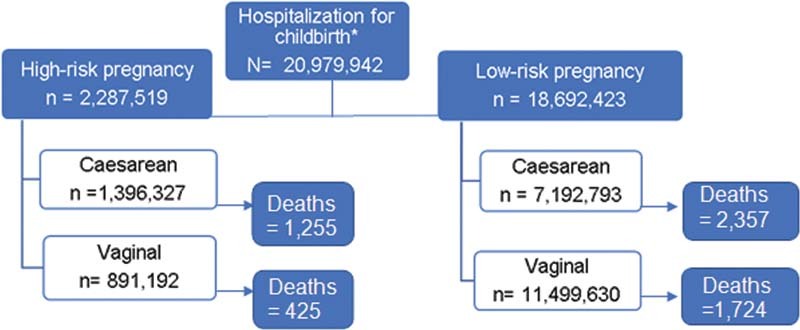
Absolute number of admissions for childbirth and maternal deaths during hospitalization according to gestational risk and mode of delivery between 2010 and 2020. *Admissions for childbirth at any gestational age were included.

The total number of pregnant women hospitalized for childbirth between 2010 and 2020 was 20,995,023, and 5,761 deaths after birth were recorded ( Figure 1 ). In 2020, the 1 ^st^ year of the COVID-19 pandemic, 1,821,775 pregnant women were hospitalized for childbirth and 651 deaths were reported, which represents 8.7% of the total hospitalizations and 11.3% of the maternal deaths in the period analyzed.

Besides, maternal mortality in high-risk pregnant women was higher than in low-risk pregnant women, in every route of delivery in the period. The average rate of maternal mortality of low-risk pregnant women from 2010 to 2019 was 2.11 (95%CI: 1.90–2.34) deaths per 10,000 admissions for childbirth, and the average rate of maternal mortality among high-risk pregnant women was 7.37 (95%CI: 6.24–8.66) deaths per 10,000 admissions for childbirth ( Table 1 ).

**Table 1.** Childbirth-related mortality rates in Brazil, according to gestational risk, and rate ratio for 2020 compared with the average for 2010-2019

	Average 2010–2019			2020			
	Hospitalization *n*	Deaths *n*	MR (95%CI)	Hospitalization *n*	Deaths *n*	MR (95%CI)	RR (95% CI)
	1915817	511	2.67 (2.44–2.91)	1821775	651	3.57 (3.31–3.86)	1.34 (1.19–1.50)
Gestational risk/Chilbirth							
Low risk	1,713,766	362	2.11 (1.90–2.34)	1554767	458	2.95 (2.68–3.22)	1.40 (1.21–1.60)
Caesarean	651,916	210	3.22 (3.09–3.36)	673633	256	3.80 (3.35–4.30)	1.18 (1.04–1.34)
Vaginal	1,061,850	152	1.43 (1.36–1.50)	881134	202	2.29 (1.99–2.63)	1.60 (1.39–1.85)
High risk	202,051	149	7.37 (6.24–8.66)	267008	193	7.23 (6.24–8.32)	0.98 (0.79–1.21)
Caesarean	122,792	111	9.04 (8.51–9.58)	168408	146	8.67 (7.32–10.2)	0.96 (0.81–1.14)
Vaginal	79,259	38	4.77 (4.30–5.26)	98600	47	4.76 (3.50–6.34)	0.99 (0.74–1.35)

Abbreviations: MR, Mortality among admissions for childbirth: number of deaths/number of admissions for childbirth x 10,000; RR, relative risk considering 2020 as exposed year and the average of the period between 2010 and 2019 as baseline.

**Fig. 2.**
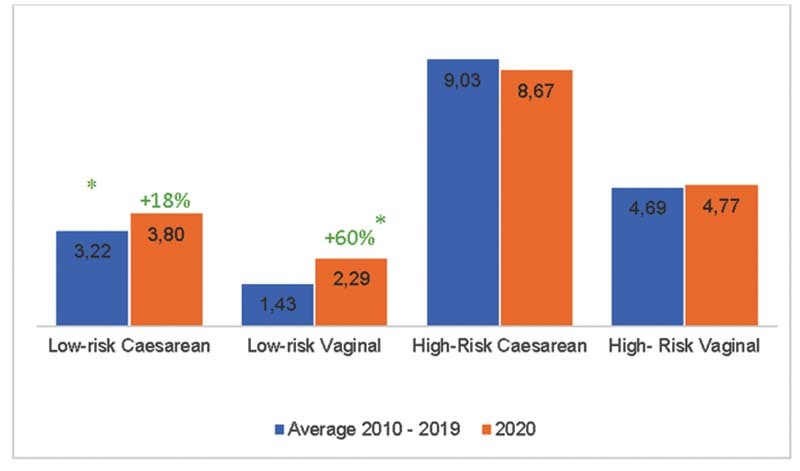
Mortality among admissions for childbirth performed in Brazil, according to route of delivery and pregnancy risk, 2010-2019 and 2020. *Statistically significant comparison

In 2020, there was an increase in maternal mortality compared with the average of the previous 10 years, with an increment of 40% in low-risk pregnancies. Women with low-risk pregnancies who underwent vaginal delivery had a 60% (RR = 1.6; 95%CI: 1.39–1.85) higher risk of dying in 2020, while those who underwent cesarean deliveries had an 18% higher risk of death (RR = 1.18; 95%CI: 1.04 -1.34) in 2020 when compared with the average between 2010 and 2019 ( Figure 2 ).

Regarding high-risk pregnancies, no significant differences were observed in mortality after vaginal or caesarean delivery in high-risk pregnancies women in the studied period.

## Discussion

The present study explored the maternal mortality among admissions for childbirth in the period between 2010 and 2020, focusing on the differences of 2020 compared with a 10-year baseline period. Higher mortality rates in high-risk pregnant women compared with low-risk is a known fact, and the association with cesarean delivery can occur because the conditions of high-risk pregnancy can be configured as indications for surgical delivery. 11 12 13 Considering this prior knowledge, the increased deaths of low-risk pregnant women, regardless of the route of delivery performed, is remarkable when comparing the period between 2010 and 2019 with the year 2020.

The increase in maternal mortality only in low-risk pregnancies allows us to suggest that the COVID-19 pandemic has been categorized as a threat to the life of this group not known until then, considering that this is a challenging situation for general health, and especially in this group with a higher immune vulnerability.

While maternal mortality in high-risk pregnancies has permanently high rates, mainly due to hemorrhage, sepsis, and hypertensive disorders, low-risk pregnant women have considerably lower mortality rates. 11 14 Thus, the COVID-19 pandemic stood out as a more dramatic and relevant harm to mothers who did not have a previous risk factor of an adverse outcome.

It is important to emphasize that, during the pandemic, prenatal care was highly affected by the fear of pregnant women to seek assistance and by barriers imposed by health care facilities for women. 15 16 The focus on the care of COVID-19-symptomatic individuals in primary care services resulted in the late arrival at hospitals of pregnant women with more serious conditions, which could have been avoided with timely and quality prenatal care. 17 In this context, high-risk pregnant women have become a priority in prenatal consultations, since many health services have changed the management of care for pregnant women, reorganizing the flow and using risk classification screening in order to focus on care for COVID-19 patients. 18 Therefore, the restriction of prenatal care, especially for low-risk pregnant women, as an indirect consequence of the COVID-19 pandemic, leads to losses in the treatment of maternal nutritional deficits, in screening for infections, and even in the classification as high-risk pregnancy if necessary, and may lead to worse maternal outcomes in this group. 19

In addition, high-risk pregnant women, aware of their most vulnerable health condition, receive greater medical guidance and, associated with the fear of infection with their risk condition, may have followed social isolation and hygiene measures more intensively and, therefore, being less frequently a target of infection and death by COVID-19. 20

However, it is a fact that, in Brazil, puerperal death by COVID-19 is mainly related to chronic problems in women's health, such as lack of resources, obstetric violence, insufficient beds, and poor-quality prenatal care. 16 Thus, the Brazilian health system was not prepared for all pregnant women to become “high-risk pregnant women” because of COVID-19, requiring greater attention and assistance while the health system was already overloaded. A study carried out with 978 Brazilian pregnant and postpartum women agrees with the present study in concluding that 51.6% of the women who died due to COVID-19 infection did not have comorbidities or risk factors. Besides, the study showed that being black, living in a periurban area, not having access to Family Health Strategy or living > 100km away from the notification hospital were associated with an increased risk of a worse outcome. 9

Therefore, the precarity of care for pregnant women and the structural racism in pandemic times may have had more impact on the deaths of postpartum women in Brazil than the association between COVID-19 infection and their previous comorbidities. A Brazilian study reinforces the insufficiency of the health system in women's healthcare by showing that 20% of pregnant and postpartum women hospitalized with COVID-19 did not have access to the intensive care unit and (ICU), and a third of them did not have access to mechanical ventilation. 4 An article published in July 2020 in The Lancet has already predicted higher maternal mortality in low- and middle-income countries due to the indirect effect of COVID-19, resulting in lower access to healthcare and to food due to the reorganization around COVID-19. The study found that 60% of additional maternal deaths would be related to basic management of women's healthcare as clean birth environments, parenteral administration of uterotonics, antibiotics, and anticonvulsants. 21 Thus, healthy pregnant and postpartum women who would need minimal assistance ended up being victims of the disorganization of the health system in pandemic times and lost their lives.

Since vaccination was recommended primarily for high-risk pregnant women, this scenario can still be maintained in 2021. 22 Thus, the mortality rates already predicted for high-risk pregnant women would remain the same, regardless of infection by COVID -19, and would increase mortality in low-risk pregnancies, since they were not yet immunized, and the possible infection would add risk to the pregnancy of these women. Therefore, the present analysis is considered preliminary and further studies with a detailed analysis of 2021 are needed to compose a more complete analysis of the pandemic period.

Finally, the increase in maternal mortality among admissions for childbirth occurred both after cesarean delivery and after vaginal delivery. However, it is noteworthy that there was a greater increase after vaginal delivery (60%) compared with cesarean delivery (18%). The literature is conflicting in assessing if the mode of delivery interferes in the maternal mortality during COVID-19 infections. A systematic review of 11,758 pregnant women found that the majority of COVID-19-infected women who died had a cesarean section (58.3%), while 25% had vaginal delivery and 16,7% of the patients were not full-term. 23 However, other systematic reviews have not found significant outcome effects comparing modes of delivery. 24 25

As for the limitations of the present study, since it is an ecological and exploratory study, comparing 1 year to a period of 10 years, a cause-effect relationship cannot be defined. Also, because of the availability of the data, we considered in comparisons only 1 year (2020) as the risk from the pandemic and did not evaluate the complete trend over the years. In addition, there are limitations inherent to the use of secondary data, depending on the quality of the record carried out and on the impossibility of evaluating the cause of death, as well as the sociodemographic profile of pregnant women, since these data are not available for analysis. Besides, there are few studies on the mortality after birth in hospitalized women, which made the comparison with the literature difficult. However, based on the increased maternal mortality of low-risk pregnancy in Brazil in 2020 compared with the period between 2010 and 2019, it is suggested that this is another unfavorable outcome of the COVID-19 pandemic in the country, since this group was characterized as a risk group for infection.

## Conclusion

Maternal mortality among admissions for childbirth according to SUS data increased in 2020 in low-risk pregnancies compared with the average number of deaths between 2010 and 2019. The increase was of 18% after cesarean section and of 60% after vaginal delivery. Regarding high-risk pregnancies, no significant differences were observed. The increase in maternal mortality only in low-risk pregnancies suggests that the COVID-19 pandemic stood out as a more dramatic and relevant harm to mothers who did not have a previous risk factor of an adverse outcome. Since puerperal death due to COVID-19 in Brazil is mainly related to chronic problems in women's health, the precarity of care for pregnant women in pandemic times may have had a greater impact on deaths among admissions for childbirth in Brazil than the association between COVID-19 infection and their previous comorbidities.
